# Practical Application of QR Code Electronic Manuals in Equipment Management and Training

**DOI:** 10.3389/fpubh.2021.726063

**Published:** 2021-11-22

**Authors:** Lifang Ma, Yonghong Mu, Ling Wei, Xiumei Wang

**Affiliations:** Shanxi Bethune Hospital, Shanxi Academy of Medical Sciences, Tongji Shanxi Hospital, Third Hospital of Shanxi Medical University, Taiyuan, China

**Keywords:** QR code, manual, operating room, equipment, training

## Abstract

**Objective:** To explore the application value of QR code electronic manuals in operating theater equipment management and training.

**Methods:** The control group adopted the traditional management mode. Training was carried out before each device was put into use in the department. A unified operation process and a paper card manual for common faults were formulated and hung next to each device. For the observation group, the electronic manuals generated by the QR code were pasted on the operating theater equipment for management and training. The management and training effect was compared between the two groups.

**Results:** The efficiency of equipment management and training using the QR code electronic manuals was significantly higher than that of traditional training, and the difference was statistically significant (*P* < 0.05).

**Conclusion:** The QR code manuals are very effective in operating theater equipment management and training. They can be read and learned in time to improve the operating theater equipment utilization rate, accuracy rate, and equipment management and training quality.

## Introduction

The QR codes are information carriers and are currently widely used. They have the advantages of a high recognition rate, a large amount of stored information, low cost and, simple operation (1). The information can be obtained by directly scanning the code with a mobile phone. The research of Guo and Xing showed that QR codes have feasible applications in medical device management (2, 3).

Operating theaters involve a complex range of surgical equipment and instruments. With the continuous improvement of surgical technology, equipment and instruments are frequently updated. In addition to mastering the needs of specialized surgery, operating theater nurses must quickly become proficient with many sophisticated kinds of equipment to ensure the smooth completion of complicated operations. The quality management, effectiveness, and safety of operating theater equipment and instruments in use is becoming more and more important (4, 5). With the improvement of the precision of the instruments and their more frequent replacement, nurses of the operating theater need to constantly update their relevant knowledge, which brings great challenges (6).

Xu's research pointed out that with the introduction of laws and regulations related to medical equipment in China (7), the quality management of medical equipment has become an inevitable trend (8). This study took the operating theater equipment and instruments purchased by the ‘136 Medical Revitalisation Project' of our hospital as the research object, and analyzed the equipment management and training involving the modern information technology of QR code electronic manuals. The aim was to grasp the use and functions of operating theater equipment and instruments in a faster and better way, give full play to the value of QR code electronic information, increase the rate of correct use of operating theater equipment and instruments, expand the scope of training, and improve the quality of hospital management.

## Materials and Methods

The application value of QR code electronic manuals was researched by comparing the management efficiency and training effect of the survey object on the two groups of equipment. The SPSS statistical software was used for data analysis, and the *t*-test was used. *P* < 0.05 was regarded as the statistically significant difference.

### General Information

Our hospital is the largest and most comprehensive Grade-A hospital investment constructed by the Shanxi Provincial Government in 2011, integrating six functions in one: medical treatment, teaching, scientific research, prevention and protection, first aid, and rehabilitation. At present, there are 30 central operating theaters and 86 operating theater teams, including 72 operating theater nurses, 13 nurse assistants, one DSA technician, and three head nurses. As of 2020, there are more than 500 fixed assets of operating theater equipment with a value of more than 60 million yuan. This study was divided into a control group and an observation group.

#### Control Group

During the equipment management period from February 2018 to January 2019, the traditional management mode was adopted. All staff were trained before the equipment was put into use, a unified operation process was formulated, and paper card manuals for common faults were hung next to the equipment and instruments according to Regulations on the Supervision and Administration of Medical Devices (revised and adopted at the 119th executive meeting of the State Council on 21 December 2020).

#### Observation Group

In September 2018, the provincial government invested 1.2 billion yuan to implement the “136 Medical Revitalisation Project” to improve the overall medical level of our province. From February to December 2019, a large amount of “136 Medical Revitalisation Project” high-precision operating theater equipment was introduced, including three different brands of 3D laparoscopic equipment, 4K laparoscopic equipment, ultrasonic suction knives, operating tables, ultrasonic knives, intraoperative B-ultrasound instruments, etc. QR code electronic manuals were used in the management and training of the equipment.

#### Survey Subjects

The 24 heads of the 12 specialist groups involved in the department's management and five nurses with less than 2 years of work experience were selected as the survey subjects. They were the main people responsible for the use of this medical equipment. To exclude the fact that experienced nurses had already been able to skilfully use the equipment of the control group, only nurses with less than 2 years of work experience were selected to directly compare the advantages and disadvantages between the two groups of equipment training management modes.

The equipment management and training results of the control group and the observation group were compared.

### Methods

The control group adopted traditional management and training. The equipment group and specialist group were responsible for management, regular inspection and maintenance, and maintaining the statistics of equipment use according to the “Equipment Use Record Book.” After the equipment was brought into the department, the head nurse in charge contacted the equipment department and engineers to train the general practitioners, and they hung the paper instructions, the simple fault causes, and treatment methods beside the equipment.

The observation group adopted the electronic manuals of the QR code, requiring suppliers and manufacturers to provide the following content before the equipment was brought into the department: (A) Company profile; (B) Equipment component icons; (C) Product advantages; (D) Usage method; (E) Troubleshooting; (F) Contact information of the engineer. For each item of equipment, they established the data source, including code, name, specification model, price, start time for usage, storage area, etc., and uniformly converted the information into a QR code, printed it onto a waterproof label, and pasted it on the equipment or instrument so that everyone could scan the code to view the corresponding information.

**Figure 1:**
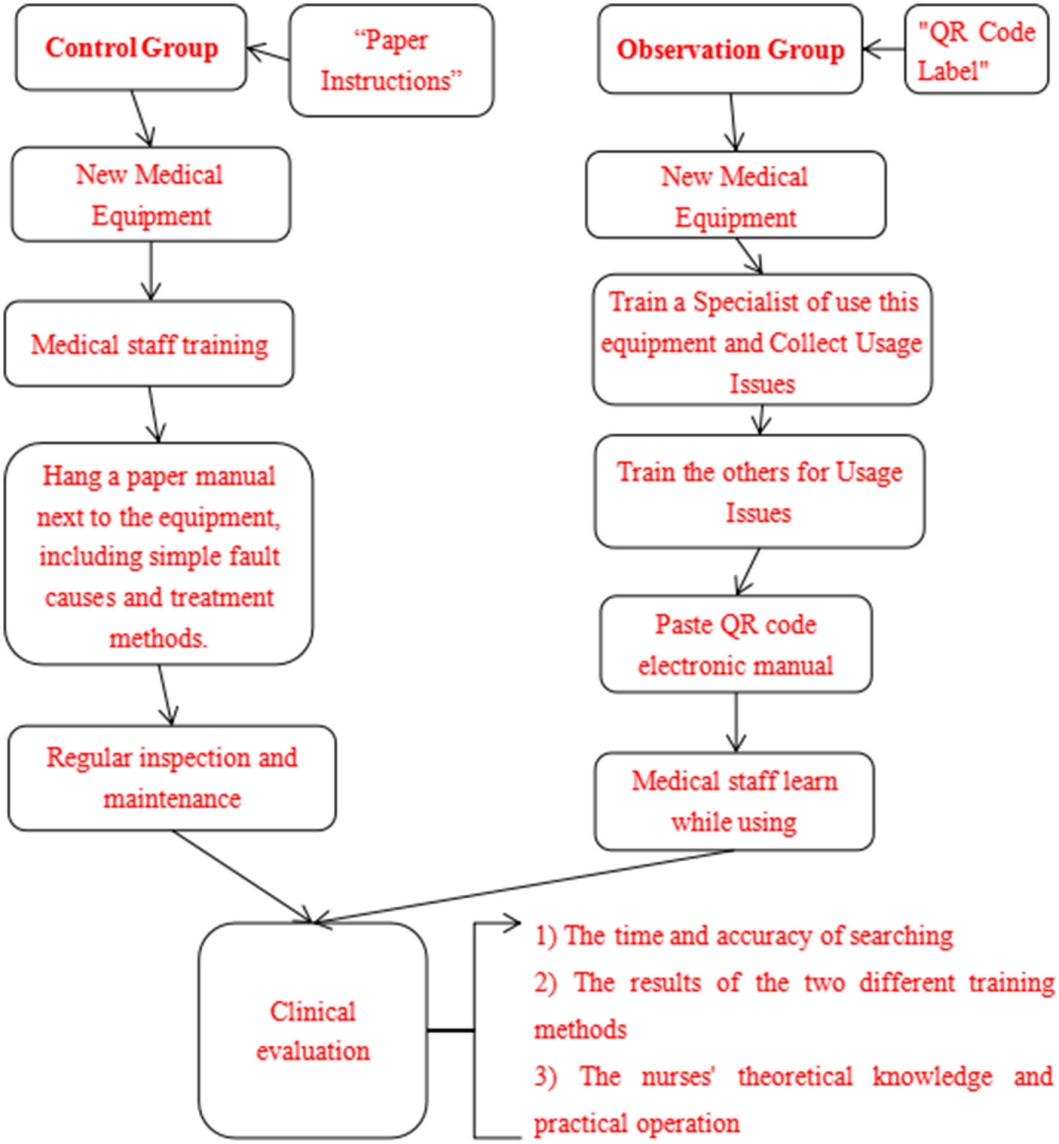
The training flow chart of the control group and the observation group.

The equipment training of the observation group followed these procedures: First, the members of the specialist team were the main training targets, and each item of equipment was managed by a specially assigned person. When the frequency of use increased, their use would be gradually promoted. Second, the specialist team collected the problems found during the use of the equipment, and in response to these, equipment engineers, doctors, and nurses were invited to conduct intensive training. They learned and solved problems together. Finally, the QR code electronic manuals were pasted, so that doctors, nurses, trainees, interns, etc. could view detailed explanations of equipment training anytime and anywhere, and the learning content could be stored permanently. The training flow chart of the control group and the observation group is shown in Figure 1.

### Clinical Evaluation

The equipment management efficiency and training results of the two groups were compared. (A) The time and accuracy required in searching for the same kind of designated equipment by the same nurse using the traditional method and the QR code-assisted method were compared. The control group first found the fixed asset code of the equipment, and then obtained the corresponding matching details, whereas the observation group obtained the relevant data by just scanning the QR code. (B) Different training methods were adopted. The control group applied for training by professional engineers immediately after the equipment was brought into the department, and the observation group followed the standard QR code manuals after the equipment was brought into the department. The results of the two training methods were compared. (C) The nurses who used the equipment scored the management and training satisfaction from the aspects of standard operation of the equipment, troubleshooting content, inventory of equipment accessories, accurate homing, statistical inquiry, learning methods, mastery degree, equipment utilization rate, etc. using the Likert A 5-level scoring method, where a score of five points means very good and a score of one means very bad (9). The theoretical knowledge of the nurses and their practical operation were assessed, and they were scored according to their mastery of the key knowledge and key steps, with a full score of 100. A higher score indicated that the nursing staff have better theoretical knowledge and operational ability of equipment management. Four types of instruments were selected for the evaluation of the time spent on searching for the equipment: laparoscopic equipment, operating tables, electrosurgical knives, and ultrasonic knives.

## Results

Using QR code electronic manuals in operating theater equipment management and training greatly improved equipment search time and accuracy, and the training effect was better than that of the control group. The operation ability of the specialist group and the nurses with different seniority on the equipment were significantly improved, the utilization rate of equipment and the satisfaction of doctors were improved, and the cooperation between doctors and nurses became better. The use of electronic manuals reduced the mess or loss of traditional paper card manuals. They were quick and convenient to produce at low cost, which reduced labor and time-management costs, and improved the learning enthusiasm of medical staff.

The satisfaction of the nurses in the observation group on the management and training of the equipment was higher than that in the control group, and the difference was statistically significant (*P* < 0.05). See Table 1.

**Table 1:** Comparison of nurses' satisfaction with instrument management and training between the two groups (points, $\bar{\text{X}}$ ± S).

**Item**	**Control group**	**Observation group**	***t* value**	***P* value**
Standard operation of the instrument	3.03 ± 0.718	4.13 ± 0.776	−12.535	0.000
Troubleshooting content	3.13 ± 0.629	4.27 ± 0.740	−12.234	0.000
Inventory of instrument accessories	3.30 ± 0.535	4.40 ± 0.675	−12.535	0.000
Accurate homing	3.43 ± 0.504	4.47 ± 0.571	−17.696	0.000
Statistical query	3.17 ± 0.531	4.27 ± 0.640	−7.940	0.000
Learning method	3.73 ± 0.640	4.60 ± 0.498	−8.308	0.000
Mastery	3.63 ± 0.556	4.37 ± 0.669	−5.809	0.000
Equipment utilization	3.30 ± 0.535	4.20 ± 0.664	−9.000	0.000

The assessment results of equipment theory and operation of the observation group were higher than those of the control group, and the difference was statistically significant (*P* < 0.05). See Table 2.

**Table 2:** Comparison of the assessment results of equipment theory and operation of the nurses between the two groups (points, $\bar{\text{X}}$ ± S).

**Group**	**Theory assessment**	**Operation assessment**
	**score**	**score**
Control group	82.77 ± 3.401	90.07± 2.273
Observation group	87.40 ± 3.918	92.33 ± 1.126
*t* value	−7.358	−5.613
*P* value	0.000	0.000

The time spent on searching for instruments and equipment in the observation group was shorter than that spent in the control group, and the difference was statistically significant (*P* < 0.05). See Table 3.

**Table 3:** Comparison of the time spent on searching for operating room equipment between the two groups (min, $\bar{\text{X}}$ ± S).

**Group**	**Laparoscopy equipment (15 sets)**	**Operating table (15 sets)**	**Electric knife (15 pieces)**	**Ultrasonic knife (15 pieces)**
Control group	21.16 ± 2.075	19.36 ± 1.753	18.24 ± 1.739	17.16 ± 1.028
Observation group	5.44 ± 1.083	5.16 ± 0.898	4.88 ± 0.726	4.56 ± 0.821
*t* value	26.012	27.922	27.485	37.093
*P* value	0.000	0.000	0.000	0.000

## Discussion

### The Application of QR Code Electronic Manuals Reduces Management Costs and Improves Management Efficiency

With the continuous development of medical technology, operating theater equipment is constantly being updated. Nurses need to master the use of various new and old items of equipment. It is of great significance to find better equipment training and management methods. The traditional management mode uses manual entry and inventory entry, which are inefficient and error-prone. It is necessary to carry out more effective equipment management (10).

The quality of equipment management in the operating theater directly affects the efficiency of the operation (11). The control group in this study adopted the traditional management mode, and the observation group used QR codes to integrate the equipment into the information management, so that the head nurse could dynamically grasp the current status and operation of various items of equipment, and nurses at all levels could check the equipment description at any time and improve the efficiency of equipment management.

There are many benefits of using QR codes. They can record a variety of text and information, so that different devices and personalized information can be effectively recorded. They have the ability to tolerate and correct errors. The medical staff does not need to worry about the loss of information due to damage to the QR code. The QR code has a small area and is simple to make by downloading a QR code generation tool, according to the unified directory specification, at a low cost. The QR code can provide a great convenience for equipment querying, tracking, and maintaining. It has high application value for equipment and instrument management, which reduces the labor and time costs of equipment management in the operating theater.

The popularization and the use of QR codes are major trends in the scientific management of operating theater equipment. However, attention should be paid to the use of QR codes, such as the surface of the QR code logo pasted on the device needs to be disinfected frequently and the QR code should be sealed with a film to make it waterproof and antifouling (12).

### The Electronic Manuals Are Conducive to New Forms of Learning, and the Training Effect Is Obvious

The control group applied for professional engineers for training immediately after the equipment was brought into the department. Each nurse failed to conduct clinical practice right away. When they were ready for use, the training content had been forgotten, and so the medical staff could only read the paper instructions or ask other personnel for guidance, resulting in incomplete mastery, passive learning, and low motivation. The utilization rate of equipment and instruments was not high, and the satisfaction of doctors was low.

For the observation group, after the equipment was brought into the department, the specialist equipment was assigned to the specialist team members as soon as possible to increase cohesion, and they received professional training ahead of the general practitioners. The course could fully reflect the ability of specialist nurses and guide doctors to improve their use of the equipment, and their motivation to learn also improved. Interactive learning with specialists and engineers could effectively solve problems, eliminate faults, and improve the satisfaction of the doctors. The scores of theoretical knowledge and practical operation were also improved, and the search time was reduced. The QR codes allowed nurses to use the fragmented time to learn at any time, which is more convenient and efficient (13).

Traditional paper card manuals have different specifications and sizes. When hanging on the sides of the devices, they are easily contaminated, lost, and are inconvenient to read during equipment use. QR codes are cheap, uniform in specification, small in area, and not easily contaminated or lost. The electronic manuals generated by QR codes are rich in pictures and text and contain comprehensive information (14). They are conducive to mobile phone learning by just scanning the code anytime, which results in the information being saved permanently. A wide range of training staff can avoid wasting time queuing to check a certain type of equipment.

A solid foundation is laid for the continuous improvement of equipment quality management only through refined training and education (15). With QR codes, the ability of nurses of different seniorities to deal with equipment failures has been improved, the overall cooperation of nurses in the operating theater has improved, the satisfaction of doctors has improved, and the training effect has been better when compared with the traditional mode.

To sum up, QR code electronic manuals are an innovative technology and play a useful role in the management and training of equipment in the operating theater. Compared with other similar researches (2, 3), this study shows that the QR code electronic manuals improve the rate of utilization and accuracy of equipment and instruments, while reducing the cost of department management and improving work efficiency. The operation of the high-precision equipment by operation theater nurses is no longer the bottleneck of performance, which reflects a fast way to the modern management of operating theater equipment. This ultimately improves the quality of operation theater management and provides a safe logistics support for patients. The incessant and constant technological and instrumental progress in the medical field has made it possible to increase knowledge and achieve new goals. However, the application of therapeutic innovations also affects the legal aspects related to medical liability (16). This needs further research and standardization.

In addition, this study has some limitations, such as the different levels of difficulty and professional knowledge required to use the equipment, and different medical personnel may use the same equipment at various frequencies, resulting in varied proficiency.

## Data Availability Statement

The original contributions presented in the study are included in the article/supplementary material, further inquiries can be directed to the corresponding authors.

## Ethics Statement

The studies involving human participants were reviewed and approved by the Ethics Committee of Shanxi Bethune Hospital. The patients/participants provided their written informed consent to participate in this study.

## Author Contributions

LM and YM conceived of the study. LW participated in its design and coordination. XW helped to draft the manuscript. All authors read and approved the final manuscript.

## Conflict of Interest

The authors declare that the research was conducted in the absence of any commercial or financial relationships that could be construed as a potential conflict of interest.

## Publisher's Note

All claims expressed in this article are solely those of the authors and do not necessarily represent those of their affiliated organizations, or those of the publisher, the editors and the reviewers. Any product that may be evaluated in this article, or claim that may be made by its manufacturer, is not guaranteed or endorsed by the publisher.
